# Serum DNA integrity index as a potential molecular biomarker in endometrial cancer

**DOI:** 10.1186/s13046-018-0688-4

**Published:** 2018-01-30

**Authors:** Enrico Vizza, Giacomo Corrado, Martina De Angeli, Mariantonia Carosi, Emanuela Mancini, Ermelinda Baiocco, Benito Chiofalo, Lodovico Patrizi, Ashanti Zampa, Giulia Piaggio, Lucia Cicchillitti

**Affiliations:** 1 Department of Experimental Clinical Oncology, Gynecologic Oncology Unit, IRCCS Regina Elena National Cancer Institute, Rome, Italy; 2 Department of Health of Woman and Child, Gynecologic Oncology Unit, Catholic University of the Sacred Heart, Rome, Italy; 3 Department of Biomedicine and Prevention, Obstetrics and Gynecology Unit, University of Rome “Tor Vergata”, Rome, Italy; 4 Department of Research, Advanced Diagnostics and Technological Innovation, Anatomy Pathology Unit Regina Elena National Cancer Institute, Rome, Italy; 5 Department of Research, Advanced Diagnostics and Technological Innovation, Area of Translational Research, IRCCS Regina Elena National Cancer Institute, Rome, Italy

**Keywords:** Endometrial cancer, Circulating cell-free DNA, DNA integrity index, Liquid biopsy, Lymphovascular space invasion, Hypertension

## Abstract

**Background:**

Circulating cell-free DNA (cfDNA) and its integrity index may represent a rapid and noninvasive “liquid biopsy” biomarker, which gives important complementary information for diagnosis, prognosis, and treatment stratification in cancer patients. The aim of our study was to evaluate the possible role of cfDNA and its integrity index as a complementary tool for endometrial cancer (EC) management.

**Methods:**

Alu-quantitative real-time PCR (qPCR) analysis wasprformed on 60 serum samples from preoperative EC patients randomly recruited. Both cfDNA content and DNA integrity index were measured by qPCR-Alu115 (representing total cfDNA) and qPCR-Alu247 (corresponding to high molecular weight DNA) and correlated with clinicopathologic characteristics. Lymphovascular space invasion (LVSI) was detected by hematoxylin and eosin staining. In case of doubt, LVSI status was further evaluate by immunohistochemistry using anti-CD31 and anti-CD34 antibodies.

**Results:**

Total cfDNA content significantly increases in high grade EC. A significant decrease of DNA integrity index was detected in the subset of hypertensive and obese high grade EC. Serum DNA integrity was higher in samples with LVSI. The ordinal regression analysis predicted a significant correlation between decreased integrity index values and hypertension specifically in tumors presenting LVSI.

**Conclusions:**

Our study supports the utility of serum DNA integrity index as a noninvasive molecular biomarker in EC. We show that a correlation analysis between cfDNA quantitative and qualitative content and clinicopathologic features, such as blood pressure level, body mass index (BMI) and LVSI status, could represent a potential predictive signature to help stratification approaches in EC.

**Electronic supplementary material:**

The online version of this article (doi:10.1186/s13046-018-0688-4) contains supplementary material, which is available to authorized users.

## Background

Worldwide, endometrial cancer (EC) is the second most common gynecological cancer and the sixth most common tumor among reproductive and postmenopausal women [1]. The main risk factors of EC are family history of EC and of certain gynecological diseases, alcohol consumption, lack of physical activity, and disorders characteristic of metabolic syndrome. EC is classified in two broad histologic types: type I (endometrioid) and type II (non-endometrioid) as established by the International Federation of Gynecology and Obstetrics (FIGO). Treatment of the diversity of this cancer presenting in the clinic is still not sufficiently personalized. Several studies showed that combination of both clinicopathologic and molecular parameters appear to represent an improvement over either system alone in the diagnosis, prognosis, and management of cancer.

Recent progress in the analysis of blood samples for circulating cell-free DNA (cfDNA) provides a rapid, cost-effective, and noninvasive “liquid biopsy” tool, which gives important complementary information on diagnosis, therapeutic targets and drug resistance mechanisms in cancer patients [2].

CfDNA is a highly fragmented double-stranded molecule including nuclear and mitochondrial DNA that is released in the blood stream through processes of apoptosis, necrosis, and secretion, autophagy and necroptosis [3–6].

The presence of cfDNA within the plasma was first reported by Mandel and Metais in 1948 in the blood of healthy individuals [7]. In 1965, Bendich and colleagues hypothesized that cancer derived cfDNA could be involved in metastasis [8]. Because of technological limitations, only several years later the first experimental evidence supporting that cfDNA in cancer patients does indeed contain tumor DNA was provided [9].

Cancer specific somatic genetic alterations can be detected in cfDNA [5, 10]. These biological characteristics discriminate cfDNA from normal cell-free DNA and assure cfDNA as a specific biomarker that provides personalized information to detect residual disease or monitor tumor progression during therapy. However, currently there are several technical difficulties challenging the practical application in cancer screening and clinical management, mainly because of the technical complexity and high cost associated with this kind of analysis.

The variability of cfDNA levels in cancer patients is likely associated with tumor burden, stage, vascularity, cellular turnover, and response to therapy with highest levels reported in cancer patients with advanced and metastatic disease [11–15]. However, cfDNA content is also elevated in various other disorders, such as infectious and autoimmune diseases, stroke, infarction and trauma [16], thus more specific approaches and accurate methodologies are needed to determine the source of cfDNA.

It has been shown that in necrosis DNA fragments are generated more randomly, have a size larger than 10,000 base pairs and could be distinguished from shorter fragments, with a 180–200 base pairs or multiples of this unit in length, produced by physiological apoptosis [17]. The degree of cfDNA integrity, called integrity index, is based on the ratio between long and short cfDNA fragments and has been recently proposed as a promising specific oncological biomarker because of its high sensitivity [6, 18].

In recent years, Alu-quantitative real-time PCR (qPCR) is the most common method used to detect DNA integrity [19–21]. The most commonly used primers for Alu-PCR are Alu115 and Alu247 [20, 21]. DNA integrity is generally calculated as a ratio of longer to shorter DNA fragments, or longer to total cfDNA content [20, 21].

In this study, we measured the concentration of cfDNA by direct qPCR of Alu repeats in serum samples from EC patients and healthy women and analysed the relationship between cfDNA results and clinicopathologic characteristics including hypertension, obesity, and LVSI. Then, we assessed the degree of cfDNA fragmentation by DNA integrity assay calculated as qPCR-Alu247 value /qPCR-Alu115 value of each sample to evaluate its potential as tool in EC stratification and management.

## Methods

### Patient cohort

All healthy volunteers and EC patients were recruited at the Regina Elena National Cancer Institute. We collected serum samples from 60 EC patients and as controls from 22 age-matched healthy volunteers with no gynecological conditions. Patients included in this study were not previously selected, but randomly chosen and recruited between 2014 and 2017. According to the histologic grade, we analyzed samples from 12 G1, 30 G2 and 18 G3 ECs. Table 1 depicts clinicopathologic characteristics of patients enrolled in this study.

**Table 1:** Clinicopathologic features of our cohort of 60 endometrial cancer patients

Endometrial cancer (grade)	G1	G2	G3
No of cases	12	30	18
Age: years Median (range)	57 (48–71)	57 (30–73)	61 (52–78)
BMI > 30%	41.7	45.4	28.7
Type I (%) Type II (%)	100.0 0.0	96.7 3.3	66.7 33.3
FIGO stage (2009) (%)			
IA	100.0	73.1	13.3
IB	0.0	19.2	26.7
II	0.0	7.7	20.0
IIIA	0.0	0.0	6.7
IIIB	0.0	0.0	13.3
IIIC1	0.0	0.0	20.0
IIIC2	0.0	0.0	0.0
IVA	0.0	0.0	0.0
IVB	0.0	0.0	0.0
MI > 50% (%)	0	31.1	88.3
LVSI (%)	0.0	6.6	77.7
Lymph node metastases (%)	0.0	0	17.5
Hypertension (%)	50.0	33.3	58.8
BMI ≥ 30 (%)	16.6	40	27.7

### Ethical approval

Experimental protocol was approved by the Ethics Committee of the Regina Elena National Cancer Institute (Rome, Italy) (RS: 2021/2017), and performed in accordance with the relevant guidelines and regulations. Written informed consent was obtained from all patients and healthy volunteers. Information about patients was obtained by reviewing their medical charts.

### Sample processing

Venous blood of cancer patients was obtained before surgery and before the beginning of any treatment. Blood samples were collected in Vacutainer tubes without anticoagulant and processed within 1–4 h. After collection the blood was allowed to clot at room temperature. The blood serum was separated by centrifugation at 1000–2000 x g for 10 min in a refrigerated centrifuge and stored at − 80 °C.

### Serum preparation

Serum preparation for qPCR was performed as described by Umetani et al. (20). Briefly, serum proteins which might hinder the qPCR results by binding to template DNA or DNA polymerase were deactivated by mixing 20 μL of each serum sample with 20 μL of a preparation buffer that contained 2.5% of tween 20, 50 mmol/L Tris, and 1 mmol/L EDTA. This mixture was digested with proteinase K (20 μg) solution for 50 min (Promega) at 56 °C, followed by 5 min of heat deactivation at 95 °C. After subsequent centrifugation at 10,000×g for 5 min, Supernatants were aliquoted and stored at − 80°.

### Quantitative PCR of Alu repeats

0.2 μL of supernatant was used as a template for each quantitative real-time polymerase chain reaction (qRT-PCR) using SYBR Green Master Mix (Applied Biosystems, CA, USA) followed by evaluation of the average of CT values from triplicate reactions from Real Time PCR software.

The sequences of the Alu115 primers were forward: 5-CCTGAGGTCAGGAGTTCGAG-3 and reverse: 5-CCCGAGTAGCTGGGATTACA-3; Alu247 primers were forward: 5-GTGGCTCACGCCTGTAATC-3 and reverse: 5-CAGGCTGGAGTGCAGTGG-3. A negative control (without template) was performed in each plate. All qPCR assays were performed in a blind fashion without knowledge of specimen identity. Mean values were calculated from triplicate reactions.

### Measurements of cfDNA values

The absolute equivalent amount of cfDNA in each sample was determined by use of a calibration curve with serial dilutions (15 ng-0.015 pg) of genomic DNA obtained from peripheral blood leukocytes of a healthy donor volunteer. A negative control (without template) was run in each reaction plate.

### Measurement of cfDNA integrity index

DNA integrity was calculated as the ratio of qPCR results using the 2 primer sets: qPCR-Alu247/qPCR-Alu115, where qPCR-Alu115 and qPCR-Alu 247 are the Alu-qPCR results obtained with the Alu115 and Alu247 primers, respectively. Because the annealing sites of Alu115 are within the Alu247 annealing sites, DNA integrity value would be 1.0 when template DNA is not truncated and 0.0 when all template DNA is truncated into fragments smaller than 247 bp. Because the Alu115 primers can amplify most fractions of circulating DNA, qPCR-Alu results obtained with Alu115 primers represent the absolute amount of DNA. The fit of the standard curve (*R*^*2*^) was higher than 0,99 for both Alu sequences.

### Statistical analysis

The examined variables were not normally distributed, as verified by the Shapiro–Wilk test, thus the non parametric U–Mann–Whitney was applied to perform a two-by-two comparison between the groups. The predictive capability (i.e., diagnostic performance) of cfDNA content was investigated by means of the area under the ROC (Receiver-Operating Characteristics) curve (AUC). Cut-offs were extrapolated from the curve. Likelihood ratio Chi-square and *P*-values were determined using logistic ordinal regression for the prediction of LVSI status and hypertension, given the levels of cfDNA (assessed as qPCR-Alu115 and qPCR-Alu247) and DNA integrity index values (assessed as qPCR-Alu247/ qPCR-Alu115). *P* ≤ .05 was considered statistically significant.

### Immunohistochemistry

Tumors from hysterectomy specimens taken immediately before surgery were formalin fixed and paraffin embedded. Three μm thick sections were cut from paraffin blocks using a cryostat and mounted onto histological glass slides and stained iwith hematoxylin and eosin. LVSI was diagnosed when viable tumor nests were observed within endothelial-lined spaces with or without intraluminal red cells or lymphocytes. In case of doubt, LVSI status was further assessed by immunohistochemical staining using the following primary antibodies: anti-CD31 mouse monoclonal antibody (M0823) (DAKO) and anti-CD34 mouse monoclonal antibody (NCL-L-END) (Leica). A pH 6 buffer was used for the two antibodies as antigen retrieval according to the manufacturer’s protocol. Staining was performed using an automated immunostainer (Bond-III, Leica). Stained slides were reviewed by experienced gynaecological histopathologists to confirm FIGO (2009) stage, histological subtype, grade, depth of myometrial invasion, and the presence or absence of LVSI.

## Results

### CfDNA content and integrity modulation in EC

CfDNA content evaluated by qPCR-Alu115 in G1 EC (median = 12.45 ng/ml; range 3.33–68.46) was very similar to that obtained from healthy specimens (median = 18.90 ng/ml; range 0.68–64.23), whereas a significant increase of total cfDNA content in G2 EC and G3 EC was detected (Table 2). Analysis of Alu247 values revealed an increased level of longer DNA fragments in sera of higher compared to lower EC grade (Table 2). We also calculated the serum DNA integrity index as qPCR-Alu247 value/qPCR-Alu115 value. Our results showed that cfDNA integrity was significantly higher only in G3 EC as shown by the higher ratio of long to total cfDNA (Table 2). Higher cfDNA and DNA integrity index values were detected in more advanced stages (Additional file 1: Table S1). However, this increase was not significant (*P* > 0.5).

**Table 2:** CfDNA levels in 12 G1, 30 G2 and 18 G3 endometrial cancer serum samples

	qPCR-Alu115 (ng/ml) Md. (range)	P –Value Mann-Whithey	qPCR-Alu247 (ng/ml) Md. (range)	P –Value Mann-Whithey	qPCR-Alu247/ qPCR-Alu115 Md. (range)	P –Value Mann-Whithey
G1	12.45 (3.33–68.46)	0.03	2.19 (0.43–11.07)	0.10	0.14 (0.09–0.47)	0.63
G2	28.45 (1.62–160.89)		5.87 (0.08–22.35)		0.14 (0.05–0.27)	
G3	29.03 (0.21–175.54	0.02	4.43 (0.51–36.15)	0.06	0.21 (0.10–0.96)	0.04

Median (Md), maximum and minimum (range), and Mann–Whitney *U* test for cfDNA values obtained by qPCR-Alu115, qPCR-Alu247, and qPCR-Alu247/qPCR-Alu115 (DNA integrity index) in G1, G2 and G2 EC

### Correlation between cfDNA content and inflammatory diseases in EC

**Fig. 1:**
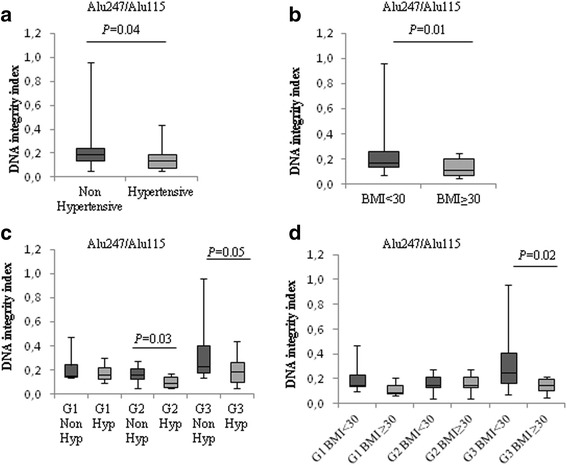
CfDNA levels related to hypertension and obesity in EC. Box-plots of DNA integrity index values in non-hypertensive and hypertensive (**a**), and normal weight (BMI < 30) and obese (BMI ≥ 30) EC patients (**b**). Cluster analysis of DNA integrity index values from non hypertensive (Non Hyp) and hypertensive (Hyp) (**c**), or normal weight (BMI < 30) and obese (BMI ≥ 30) (**d**) in G1, G2 and G3 EC samples. The upper border of the box indicates the upper quartile (75th percentile) while the lower border indicates the lower quartile (25th percentile), and the horizontal line in the box the median. *P* values, Mann–Whitney *U* test

In order to obtain an optimal cut-off that best discriminated between high (G2 and G3) and low grade (G1) EC, we performed ROC analysis by comparing values from qPCR-Alu115, qPCR-Alu247 and DNA integrity index. All three markers showed a low predictive accuracy, indicating that this method is not sufficient by itself to differentiate high grade from low grade EC patients (Additional file 2: Table S2). To assess if chronic inflammatory diseases, such as hypertension and obesity, correlated with the amount of cfDNA released in EC, we clustered samples from hypertensive and non hypertensive patients, and from patients with BMI < 30 and BMI ≥30. The percentage of hypertensive and obese EC patients is shown in Table 1. We observed a trend, even if not significant, towards higher total cfDNA levels in hypertensive and obese patients (Table 3). DNA integrity index was significantly lower in hypertensive and obese patients (Fig. 1a and b, and Table 3). Cluster analysis based on EC grading revealed that a significant down-modulation of DNA integrity index occurred specifically in samples from hypertensive and obese compared, respectively, with normotensive and normal weight high grade EC patients. (Fig. 1c and d). The logistic regression test was applied to analyse the relationship between DNA integrity indexes and hypertension and overweight. The model predicted no direct correlation (*P* > .05).

**Table 3:** Association between cfDNA measurements and blood pressure levels (non-hypertensive and hypertensive), body mass index or lymphovascular space invasion in 60 endometrial cancer patients

LSVI and blood pressure status in ECs	qPCR-Alu115 (ng/ml) Median Mean	*P* –Value Mann-Whithey	qPCR-Alu247 (ng/ml) Median Mean	*P* –Value Mann-Whithey	DNA integrity index Median Mean	*P* –Value Mann-Whithey
LSVI negative (LVSI-)	23.98 48.9	0.54	3.58 7.2	0.25	0.14 0.16	0.03
LSVI positive (LVSI+)	30.61 49.4		6.56 11.1		0.22 0.27	
Non-hypertensive	26.34 32.99	0.38	4.89 7.4	0.78	0.19 0.23	0.04
Hypertensive	42.97 53.35		4.84 12.5		0.13 0.15	
LSVI- non-hypertensive	22.05 43.7	0.58	4.43 7.06	0.70	0.14 0.17	0.77
LSVI- hypertensive	28.45 60.5		3.63 8.29		0.13 0.14	
LSVI+ non-hypertensive	29.02 39.1	0.44	6.11 8.90	0.55	0.23 0.32	0.05
LSVI+ hypertensive	71.53 66.4		14.01 14.6		0.14 0.18	
BMI < 30	20.35 37.59	0.25	5.10 8.51	0.76	0.17 0.22	0.01
BMI ≥ 30	26.96 46.47		3.53 7.13		0.11 0.12	
LSVI- BMI < 30	16.81 29.60	0.16	3.18 6.86	0.50	0.14 0.17	0.17
LSVI- BMI ≥ 30	28.45 62.66		3.63 7.78		0.11 0.12	
LSVI+ BMI < 30	32.75 51.43	0.82	6.82 12.13	0.63	0.22 0.30	0.29
LSVI+ BMI ≥ 30	26.78 43.12		4.25 7.88		0.21 0.18	

Median, mean and Mann–Whitney *U* test for cfDNA values obtained from qPCR-Alu115, qPCR-Alu247 and DNA integrity index (qPCR-Alu247/qPCR-Alu115) in samples from hypertensive and non-hypertensive patients, from patients with BMI < 30 and ≥30, and from LVSI negative (LVSI-) and positive (LVSI+) tumors

*LVSI* Limphovascular Space Invasion, *BMI* Body Mass Index

### Correlation between cfDNA integrity index with LVSI in high grade EC

**Fig. 2:**
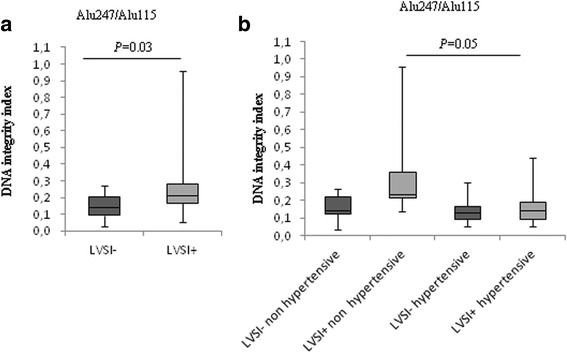
Hypertension affects serum DNA integrity indexes in LVSI positive ECs. Box-plots of DNA integrity index values in LVSI negative (LVSI-) and LVSI positive (LVSI+) serum samples (**a**), and association between LSVI status and blood pressure levels (**b**). *P* values, Mann–Whitney *U* test

We also investigated the possible involvement of LVSI in cfDNA release and integrity. LVSI was assessed by morphology and, in case of doubt, also by immunohistochemistry using anti-CD31 and anti-CD34 antibodies. We grouped serum samples from EC patients with or without LVSI. A trend toward increased cfDNA levels, evaluated by qPCR with both Alu115 and Alu247 couple of primers, was observed in samples with LVSI (Table 3). A significant increase was observed for DNA integrity index values in LSVI+ serum samples (Fig. 2a and Table 3). Cluster analysis of samples from hypertensive and non-hypertensive EC patients, or from patients with BMI < 30 and ≥30 showed that the higher DNA integrity index was encountered in LSVI+ samples derived from non-hypertensive patients. The other subgroups showed comparable values (Fig. 2b and Table 3). Based on these results, to better determine the relationship between DNA integrity index values and hypertension in LVSI + EC samples, we performed a logistic regression test for the prediction of LVSI status and hypertension, given the values of DNA integrity index. Decreased DNA integrity values significantly correlated with the hypertensive status in high grade ECs with the presence of LVSI (likelihood ratio chi square = 3.691, *P* < .05 with degree of freedom = 1).

## Discussion

EC is the most common cancer of the female female reproductive organs and is the seventh most common cause of death from cancer in women in Western Europe. At diagnosis, about 75% of women have a cancer confined to the uterus (stage I) and the prognosis is good. However the prognosis for recurrent or metastatic EC remains poor, thus more sensitive methods to help clinical diagnosis and improve the stratification of EC patients are needed.

In our study, we analysed the quantities and degree of cfDNA integrity in EC serum samples in order to assess cfDNA content as a simple and inexpensive non-invasive complementary tool for EC stratification. We performed a quantification of cfDNA by qPCR for Alu repeats using as the template, serum without preeceding DNA purification, thus overcoming artifacts associated with DNA isolation, such as the prevailing short-comings of DNA extraction methods, [20]. We used two sets of Alu primers: the primer set for the 115 bp amplicon (Alu115) that amplifies both shorter (truncated by apoptosis) and longer DNA fragments, and a set for the 247 bp amplicon (Alu247) that amplifies only longer DNA fragments. It is generally accepted that the 180–200 base pairs or fragments length of multiples of this unit reflect apoptosis, the prevalent mechanism of cell death in physiologically conditions, whereas necrosis, producing much longer DNA fragments on account of an inefficient nuclease activity, seems to occur more frequently in tumor cells [22, 23]. Is has been suggested that DNA integrity, calculated as a ratio of longer to shorter DNA fragments, specifically represents the relative amount of non physiological non apoptotic cell death.

In our study, we observed a significant increase of both cfDNA content and DNA integrity index in high grade compared with G1 ECs, suggesting a role of cfDNA as potential prognostic biomarker in EC. Conversely, in a previous study Tanaka et al. did not find significant modulation in cfDNA content among both histological EC grade or stage [24]. We suggest that the difference in the results may be due to the different procedure applied and, in particular, to prior processing of samples and DNA extraction. However, ROC curve analysis revealed a poor diagnostic power of the cfDNA assay to differentiate low grade from high grad EC.

It has been shown that levels of inflammation are directly correlated with the amount of cfDNA release [25–28]. Levels of cfDNA concentration are also associated with pathophysiology of hypertension and vascular damage [29]. Several studies have demonstrated a correlation between hypertension and obesity and the relative risk of developing EC cancer [30]. We found an inverse relationship between the presence of these two inflammatory diseases and DNA integrity values in high grade EC. It is possible to hypothesise that the levels of inflammation status contribute to a larger cfDNA release of short fragments into the bloodstream. This mechanism may very likely lead to a relative increase in short cfDNA fragments, that may explain the decreased cfDNA integrity indexes observed.

It is worthwhile to note that high blood pressure and obesity affected specifically and exclusively the degree of cfDNA fragmentation in high grade EC sera, whereas not detectable differences in G1 EC samples were measured. It has been shown that the DNA integrity index varies between different cancers and also among individual cancers [31–33]. We hypothesise that cfDNA content in G1 EC is still under the clearance capacity of phagocytic cells and that nuclease activity is still efficient, thus effects may not be detected. Moreover, by qPCR-Alu115 it is not possible to amplify very small DNA fragments, and this may affect the detection level and the yield of this technique. In fact, we could not detect differences between cfDNA content in healthy control and G1 EC group. On the other hand, in higher grade EC, the rapid DNA release from a higher number of cancer cells may overwhelm phagocytosis resulting in the increased cfDNA accumulation of fragments detectable by qPCR into circulation due to both apoptosis ans necrosis.

An important criterion for further therapy in cancer is represented by LSVI status. LVSI includes lymphatic vessel and blood vessel invasion involved in tumor spreading and metastasis by its ability to enhance dissemination of viable cancer cells and release of DNA from malignant tumor into the blood stream. LVSI had been suggested to be an important prognostic factor for relapse of disease and poor survival in patients with ovarian [34], vulva [35], cervical [36, 37], rectal [38], breast [39], ans lung cancers [40], and EC [41]. Therefore, we focused on cfDNA level related to LVSI status. Interestingly, we observed a significant increase of DNA integrity index in serum samples from EC with LVSI (LVSI+) compared to those from cancer without LVSI (LSVI-), further supporting the hypothesis that the relative higher content of longer cfDNA fragments in serum samples very likely derives from cancer cells. Interestingly, our data obtained from correlation analysis between cfDNA content and the inflammatory status due to hypertension or obesity in ECs presenting LVSI indicated that the higher DNA integrity index was specifically encountered in LSVI+ samples derived from non-hypertensive patients, further confirming a pivotal role of hypertension in affecting DNA integrity index values.

As a aperspective for EC patients, further analysis in large cohort are needed in order to better optimize the accuracy and reliability of cfDNA measurements and confirm its suitability for clinical use, taking into account a longer follow-up and specific markers of tumor origin [42–45].

## Conclusions

Our pilot study provides reliable evidence on the utility of cfDNA analysis in EC for further validation studies, with some limitations represented by the short follow up that does not allow to reach a definitive endpoint in terms of clinical utility for prognosis. For this reason, we are currently collecting longer term follow up data.

We suggest that measurement of cfDNA content in serum samples by qPCR-Alu115 and qPCR-Alu247 may represent a potential simple and not expensive molecular tool to better surgical staging and help in EC stratification. In particular, on the basis of our result on the correlation between serum DNA integrity index and LVSI, we envisage the possibility that this approach might be useful to identify high grade EC with risk of metastasis. A longer follow up is necessary to validate this hypothesis.

Finally, from a molecular point of view, a better understanding of the source and clearance mechanisms of cfDNA, such as metabolic changes in the rate of cfDNA turnover, inefficient removal of dead cells or release of DNA from tumor microenviroment and blood cells, would improve the interpretation of our study.

## Additional files

Additional file 1: **Table S1.** CfDNA content and EC staging. Measurement of median cfDNA values obtained by qPCR-Alu115, qPCR-Alu247, and pPCR-Alu247/qPCR-Alu115 (DNA integrity index) in different EC stages. (DOCX 15 kb)

Additional file 2: **Table S2.** Receiver operative characteristics (ROC) analysis and optimal cut-offs values for cfDNA evaluated by qPCR-Alu115, qPCR-Alu247 and qPCR-Alu247/qPCR-Alu115 in G2 and G3 EC versus G1 EC serum samples. (DOCX 11 kb)

## Abbreviations

BMI: Body mass index
cfDNA: Cell-free DNA
CI: Confidence interval
EC: Endometrial cancer
LVSI: Lymphovascular space invasion
qPCR: Quantitative real time PCR
ROC: Receiver operating characteristic
