# Correction to: Serum DNA integrity index as a potential molecular biomarker in endometrial cancer

**DOI:** 10.1186/s13046-018-0708-4

**Published:** 2018-02-20

**Authors:** Enrico Vizza, Giacomo Corrado, Martina De Angeli, Mariantonia Carosi, Emanuela Mancini, Ermelinda Baiocco, Benito Chiofalo, Lodovico Patrizi, Ashanti Zampa, Giulia Piaggio, Lucia Cicchillitti

**Affiliations:** 1 Department of Experimental Clinical Oncology, Gynecologic Oncology Unit, IRCCS Regina Elena National Cancer Institute, Rome, Italy; 2 Department of Health of Woman and Child, Gynecologic Oncology Unit, Catholic University of the Sacred Heart, Rome, Italy; 3 Department of Biomedicine and Prevention, Obstetrics and Gynecology Unit, University of Rome “Tor Vergata”, Rome, Italy; 4 Department of Research, Advanced Diagnostics and Technological Innovation, Anatomy Pathology Unit Regina Elena National Cancer Institute, Rome, Italy; 5 Department of Research, Advanced Diagnostics and Technological Innovation, Area of Translational Research, IRCCS Regina Elena National Cancer Institute, Rome, Italy

## Correction

In the publication of this article [1], there is an error in the first sentence of the Acknowledgements section.

The error: ‘This work has been founded by IRE Internal Projects to G. P. and E. V.’

Should instead read: ‘This work has been founded by IRE Internal Projects to G. P. and E. V. and Lazio Region BTO project to E. V.’

This has now been included in this erratum.
