# Supplementary material for: Serum DNA integrity index as a potential molecular biomarker in endometrial cancer
Source: J Exp Clin Cancer Res. 2018 Jan 30;37:16. doi: 10.1186/s13046-018-0688-4 (PMC5791183; doi:10.1186/s13046-018-0688-4)
Supplement: Supplementary file 1 — Table S1. CfDNA content and EC staging. Measurement of median cfDNA values obtained by qPCR-Alu115, qPCR-Alu247, and pPCR-Alu247/qPCR-Alu115 (DNA integrity index) in different EC stages. (DOCX 15 kb) [file 13046_2018_688_MOESM1_ESM.docx]

Additional file 1: Table S1

**CfDNA content and EC staging**

| Stage | qPCR-Alu115  Md. (range) | qPCR-Alu247  Md. (range) | DNA integrity index  Md. (range) |
| --- | --- | --- | --- |
| IA | 22.05  (1.62-160.89) | 3.55  (0.08-49.85) | 0.15  (0.05-0.47) |
| IB | 22.28  (1.08-49.82) | 4.24  (0.34-22.35) | 0.20  (0.06-0.27) |
| II | 36.33  (28.45-110.17) | 5.60  (2.82-31.18) | 0.16  (0.10-0.40) |
| IIIA | 37.66  (14.85-60.47) | 3.70  (0.53-6.86) | 0.07  (0.04-0.11) |
| IIIB | 91.59  (7.65-175.54) | 10.02  (1.74-18.29) | 0.17  (0.10-0.23) |
| IIIC1 | 33.63  (0.20-82.59) | 18.70  (1.24-36.15) | 0.44  (0,05-0.96) |

Median serum cfDNA content (Md) and maximum and minimum (range) evaluated by qPCR-Alu115, qPCR-Alu247, and pPCR-Alu247/qPCR-Alu115 in different EC stages.
