# Supplementary material for: Serum DNA integrity index as a potential molecular biomarker in endometrial cancer
Source: J Exp Clin Cancer Res. 2018 Jan 30;37:16. doi: 10.1186/s13046-018-0688-4 (PMC5791183; doi:10.1186/s13046-018-0688-4)
Supplement: Supplementary file 2 — Table S2. Receiver operative characteristics (ROC) analysis and optimal cut-offs values for cfDNA evaluated by qPCR-Alu115, qPCR-Alu247 and qPCR-Alu247/qPCR-Alu115 in G2 and G3 EC versus G1 EC serum samples. (DOCX 11 kb) [file 13046_2018_688_MOESM2_ESM.docx]

**Additional file: Table S2:** **Receiver operative characteristics (ROC) analysis and optimal cut-offs values for cfDNA evaluated by qPCR-Alu115, qPCR-Alu247 and qPCR-Alu247/qPCR-Alu115 in G2 and G3 EC versus G1 EC serum samples**

| **Receiver Operating Characteristic**  **(ROC) analysis** | Area under the ROC curve  (95% confidence interval ) | Best cut-off | Sensitivity  at the best cut-off | Specificity  at the best cut-off |
| --- | --- | --- | --- | --- |
| qPCR-Alu115 | 0.701  (0.612-0.789) | 20 ng/ml | 57.0% | 76.7% |
| qPCR-Alu247 | 0.583  (0.503-0.664) | 1 ng/ml | 46.5% | 67.1% |
| qPCR-Alu247/  qPCR Alu115 | 0.506  (0.425-0.589) | 0.4 | 42.8% | 58.1% |
